# Alveolar Ulceration Distinguished from Alveolar Abscess

**Published:** 1881-07

**Authors:** L. C. Ingersoll


### THE DENTAL REGISTER.

VOL. XXXV.] JULY, 1881. [No. 7.

### ALVEOLAR ULCERATION DISTINGUISHED FROM ALVEOLAR ABSCESS.

BY L. C. INGERSOLL.

*Read before the Iowa State Dental Society, May 10, 1881.*

It has been customary with many in the profession to use the words *abscess* and *ulceration* as synonymous terms—applying them indiscriminately to all deep-seated disease located within the alveoli of the teeth, affecting the root-membrane and surrounding parts, and accompanied with the discharge of purulent matter.

In general, the term *alveolar abscess* has been assumed to cover all modifications of deep-seated disease, while the term *ulceration* has been employed to designate a superficial disease of the gum and exposed portion of the root-membrane.

This general application of the word *ulceration* to the suppurative process on exposed surfaces should not mislead any, to think the term a misnomer when applied to the same process concealed from view in the alveoli, fauces, lungs, stomach, intestines, or on any portion of the mucus surfaces.

Diseases which are distinct from each other have anatomical and pathological peculiarities by which they are distinguished.

One year ago I presented before you the subject of *alveolar abscess* in one of its most common manifestations, and in that paper I aimed to distinguish it from *alveolar ulceration.*

In treating now upon the latter I desire to repeat the distinctions then made, alluding to that *deep-seated* ulceration so often mistaken for alveolar abscess.

The following are the most manifest differences:

1st. An abscess has a fibrous sack in which the pus is confined; an ulcer has no sack.

2d. An abscess has a fistulous tube or canal leading out from the abscess sack to the surface of the mucus membrane or to the surface of the skin, through which the confined pus is discharged. An ulcer has no such tube, but the purulent matter formed oozes out through the gum, or around the neck of the affected tooth.

3d. In the development of an abscess new tissue is formed. In the development of an ulcer normal tissue is disorganized and wasted.

4th. The matter discharged from an abscess is thick and opaque, and of an offensive odor; that discharged from an ulcerous condition is watery, translucent, and in many cases quite odorless.

5th. An abscess manifests itself by frequent swelling; ulceration seldom, if ever, causes swelling.

Both are alike in creating a cavity about the root which gives room for the expansion of the abscess or the ulcer; but they are unlike in the causes operating to produce the cavity. In the case of abscess the cavity is formed by a physiological process; in the case of ulceration, by a pathological process. In the case of abscess, pressure of the accumulating pus excites into action the natural function of absorption, which produces the cavity, and also makes the way out through the alveolar wall for the fistulous tube. In the case of alveolar ulceration the inflammatory action is phagedenic in its character—wasting away both hard and soft tissue by chemical decomposition.

Alveolar absess and alveolar ulceration having so many points of difference, embracing all their most observable manifestations, cannot be regarded as the same disease, or as different stages of developmental progress of the same disease. We find hundreds of cases of true alveolar abscess, which, though having existed for years, have never arrived at the pathological condition which I have described as *alveolar ulceration*.

Nor are their differences harmonized by the fact that abscess and ulceration have usually a common origin—that of the death of the pulp. Catarrh and consumption may arise from the same cause—taking cold—yet they are not the same disease. Nor is the fact that the disease which begins as abscess may terminate in ulceration any evidence of the identity of the two diseases; no more so than that the disease which begins as catarrh and terminates in consumption, is evidence that these two diseases are identical—only in different stages of progress.

I do not regard alveolar ulceration a primary disease. It is always, I believe, preceded by some other well established and usually chronic disease.

When an exposed pulp dies a slow spontaneous death, there usually happens as a result a chronic inflammation, or a tumefaction of the peridental membrane, or alveolar abscess.

In case a tumor be formed on the root-membrane, it may change to an abscess or to an ulcer. Ulceration is not an unusual termination of a tumor; it is one of Nature's modes of effecting a cure, and, under favorable circumstances, does effect a cure. But this favorable termination is not usual with an alveolar tumor. At the same time, the change of the character of the disease from a tumor to an ulcer renders the disease more accessible and more easily cured.

An abscess, too, in a chronic condition, may change to an ulcer; but instead of rendering the case more favorable for treatment and cure, the metamorphosis increases the difficulty, as judged by my own experience. When pus begins to form about the root of a tooth, the ordinary functions are ample for disposing of it by absorption; but when the amount of pus increases beyond this functional capacity for removal, farther provision is made in the formation of a pocket lining the abscess cavity, and a tube-drain to evacuate the pus out upon the surface. Should the benign character of the pus change to an acrid condition with its concomitants, we may expect this whole morbid functional apparatus employed in the evacuation to be broken up, the abscess lining penetrated, the pus escaping and infiltrating into the surrounding tissues. The capillary vessels become involved in the inflammatory condition, and *liquor san-*

*guinis* is freely poured forth as one of Nature's modes of liquifying the pus and facilitating its escape through the tissues. This is now the ulcerated condition. The pus is made up of tissue-debris, *liquor sanguinis* and emigrant blood corpuscles. It differs in consistency in proportion to the amount of insoluble tissue-debris contained. In its most benign character it is little else than *liquor sanguinis*—the solid portions of the blood remaining behind in the veins and capillaries in the form of coagula. This peculiar feature in the formation of the ulcerative state, and known pathologically as *thrombosis*, is well worthy of attention.

Thrombosis is the formation of coagula in veins and capillaries of the living subjects similar to the coagula formed after death. In the examination of the physical organism we are struck with the ample provision against the destruction of tissues and of life.

The formation of thrombi seems to be a provision of Nature against the destruction of the vascular system and the extensive impairment of nutrition by the extravasation of the blood. When a blood vessel becomes irritated and its membranous coatings inflamed, this is a signal of danger, and is met by a determination of blood at that point. The white blood corpuscles, that travel in close succession along the walls of the vessel, arrange themselves and become glued together in a compact layer about the affected part, and upon this a coagulum of blood is formed, and thus ultimately the thrombus is made up of successive layers of white corpuscles and fibrinous coagulum, which would completely stop the flow of blood from any opening that might be made by the inflammatory process through the walls of the vein, and also would prevent the influx of poisonous pus among the living tissues. Were it not for this formation of thrombi, extravasated blood might be found in great profusion wherever inflammatory action had progressed so far as to break through the vascular tissues. In the formation of this coagulum the liquor sanguinis is liberated, just as in the coagulation of milk the whey becomes separated from the casein.

In the formation of an alveolar ulcer we have just the conditions which I have noticed above. All the inflammatory processes which precede ulceration are irritating and destructive to the vascular tissues that surround an abscess. On the formation of thrombi the watery portion of the blood is transuded, to be thereafter absorbed, or form serous pus. As the process of vascular repair goes on and new vessels are formed, the thrombi are no longer needed to prevent the extravasation of blood, and, if re organization of the thrombi into new tissue does not take place by a slow process, they become softened into pus, and mingle with the transuded serum. There is such a breaking up of tissues in the region of an alveolar ulcer that an examination will often show the destruction of a considerable portion of the root-membrane, the walls of the alveolus over the same part, the cementum, and, possibly, a wasting of the dentine.

If it has been preceded by alveolar abscess, as it usually is, the lining of the abscess cavity becomes broken up, and the fistulous canal also. Though the sinus upon the gum may remain as a mouth for the discharge of pus, it is probable that the soft tissue surrounding the abscess canal very soon disappears after the ulcerative condition has been established; and a probe entering, would come directly in contact with either the alveolar bone or tooth bone. Having before us this general view of the destructive processes of alveolar ulceration, the question of cure and restoration to soundness, of the parts involved, is formidable. Such teeth often remain in this condition for years, giving no pain or trouble to the patient which is sufficiently annoying to cause him to seek relief.

The cure of alveolar abscess is simple and rapid; and the restoration both in substance and function is complete, except the pulp, which is lost beyond recovery. We have the root-membrane intact, only in an inflamed and morbid condition; the alveolar walls firm and free from disease, only perforated at a single point to permit the drain-tube to pass through. We have an abscess cavity to be refilled with healthy tissue, but we also find present all the functional powers for the repair work so soon as the abnormal tissue composing the sack or cavity-lining is removed. A cure is therefore effected by a thorough breaking up and disposal of the abscess sack.

In case of ulceration, if it has existed for a considerable length of time, it is not simply the tooth pulp that is gone, as in case of abscess, but several kinds of normal tissue are destroyed. A cure means the restoration of all these lost tissues except the pulp. Whether they will be restored depends as much upon constitutional diatheses as upon therapeutical treatment. This fact throws around the case difficulties which must enter into a correct diagnosis. A case may require constitutional as well as local remedies. In laboring over a perplexing case we may find necrosis. This, also, would complicate the case, and make treatment both tedious and severe.

Another complication, which recently came under my notice in a case presented for treatment, is worthy of notice. The tooth was affected both with *abscess* and *ulceration*. These were wholly independent of each other—there being no communication between the two. The ulceration was located beneath and around the crater of an old abscess that had run its career about five years previous.

Since the breaking up of the abscess and the change in the character of the disease there had been no swelling and no pain except sufficient barely to call attention to the locality. The discharge from it for years past had been but a watery fluid, with none of the disgusting appearance of pus from an abscess.

This ulceration was located about midway between the apex of the root and the margin of the gum.

The root of the tooth having remained open all this while, had, from the irritating nature of accumulations within, a second time caused the inflammatory condition and full development of abscess, with the usual swelling. Instead, however, of discharging through the old sinus where ulceration existed, it formed a new fistulous opening, which, as before remarked, had no communication with the ulcer. The abscess was readily cured. The ulceration I still have under treatment. This is the first and only case I have ever met with, troubled at the same time with both diseases—declaring most fully their distinctive differences.

Another of the peculiar symptoms often present in cases of alveolar ulceration of long standing, is the formation of granules of tartar on the roots—sometimes in continuity of particles, and sometimes in separate granules, extending along the line from the point of ulceration to the margin of the gum.

This ulcerative condition and deposit of tartar should not be confounded with the disease known as *pyorrhœa-alveolaris*, sometimes called Rigg's Disease. In the latter case the calcareous deposit is the *cause*, in the former the *result*, of the ulcerative condition.

Wherever this deposit is found, whether at the neck of the tooth or at the apex, it is a foreign body acting as an irritant, and must be removed before a cure can be effected. The removal of this deposit must be accomplished by mechanical and chemical means.

The root of the tooth should first be scraped over the entire ulcerative surface. Then, as there may be particles missed in the operation, a dilute acid should be used to dissolve whatever portion of the deposit may remain. A cure depends upon the thoroughness of the removal of the deposit, if existing. In some cases the operation is extremely difficult, requiring careful manipulation and persevering effort.

In the distinction which I have made between this deep-seated ulceration and that which is caused by salivary calculus deposited about the necks of teeth, I have given intimation of a new source of calcareous deposit upon the teeth other than the saliva.

All lime deposits in the mouth having any connection with the teeth have been called salivary calculi, because of their supposed salivary origin. That the tartar so visibly common in so many mouths, is a deposit from the saliva, is too evident, I think, to admit of dispute.

But the tartar brought to your notice as formed on the roots of teeth at and near their terminal points, is located *beyond the reach of saliva*, and cannot therefore be referred to the saliva as its source, nor can it properly be called *salivary calculus*.

All the conditions connected with the form of ulceration now under consideration, indicate that *serum* is the immediate source of the calcareous deposit. And as the serum flowing freely from an ulcerating surface is a transudation from the blood, and more technically known as *liquor sanguinis*, the blood itself must be regarded the real source of the deposit. I have, therefore, named it *sanguinary calculus;* thus indicating its origin. That this is possible and altogether demonstrable, will appear from a consideration of a few facts.

The blood contains the entire list of nutrient elements composing the body. The blood holds in solution all the lime salts that enter into the formation of bone, and the hard tissues of the teeth. When, then, *liquor sanguinis* is separated from the insoluble portions of the blood, as in case of ulceration, it carries with it these mineral elements, which coming in contact with the lime salts composing the tooth substance, by the law of affinity, the lime in the *liquor sanguinis* parts with that fluid, and attaches itself firmly to the root of the tooth.

Further, it should be noticed that the salivary glands secrete the saliva from the blood; so that what lime is found as a constituent part of salivary calculus, must be referred to the blood as its primary source. The same is true of the glandular system generally—whatever fluid the glands secrete to mingle with other elements comes from the blood, and holds in solution the same lime salts as elsewhere found; hence we have *biliary calculus* and *urinary calculus.* Calcification of valves of the heart, and calcification of veins and arteries, must be referred to the same source. The lime deposit in connection with the ulcerative process coming so directly from the serum of the blood, without chemical change and without the intervention of glands or other secretive organs, may, with greater propriety than calcareous deposits elsewhere found, be called *sanguinary calculus.*

Returning now from this slight digression, I wish to direct special attention to its frequent occurrence in cases of ulcerated teeth of the class described, and also to the necessity of its entire removal as a means of cure. No doubt, many cases have baffled the skill of other dentists, as they have my own, without the operator suspecting the true cause of the failure. I desire to commend this subject as now presented, to your thoughtful and studious attention.

To observe distinctions founded on radical differences, is one of the laws of correct diagnosis; and a correct diagnosis is a prerequisite to successful cure of disease.

